# Synergistic Network Pharmacology for Traditional Chinese Medicine Liangxue Tongyu Formula in Acute Intracerebral Hemorrhagic Stroke

**DOI:** 10.1155/2021/8874296

**Published:** 2021-02-26

**Authors:** Yang Chen, Ju Dong, Dongqing Yang, Qin Qian, Pengcheng Wang, Xiaojuan Yang, Wei Li, Guochun Li, Xu Shen, Fushun Wang

**Affiliations:** ^1^Department of Public Health of Nanjing University of Chinese Medicine, 210023 Nanjing, Jiangsu Province, China; ^2^State Key Laboratory Cultivation Base for TCM Quality and Efficacy, Nanjing University of Chinese Medicine, 210023 Nanjing, Jiangsu Province, China; ^3^Institute of Brain and Psychology, Sichuan Normal University, 610060 Chengdu, Sichuan Province, China

## Abstract

**Background:**

Nowadays, acute intracerebral hemorrhage stroke (AICH) still causes higher mortality. Liangxue Tongyu Formula (LXTYF), originating from a traditional Chinese medicine (TCM) prescription, is widely used as auxiliary treatment for AICH.

**Objective:**

To dig into the multicomponent, multitarget, and multipathway mechanism of LXTYF on treating AICH via network pharmacology and RNA-seq.

**Methods:**

Network pharmacology analysis was used by ingredient collection, target exploration and prediction, network construction, and Gene Ontology (GO) and KEGG analysis, with the Cytoscape software and ClusterProfiler package in R. The RNA-seq data of the AICH-rats were analyzed for differential expression and functional enrichments. Herb-Compound-Target-Pathway (H-C-T-P) network was shown to clarify the mechanism of LXTYF for AICH.

**Results:**

76 active ingredients (quercetin, Alanine, kaempferol, etc.) of LXTYF and 376 putative targets to alleviate AICH (PTGS2, PTGS1, ESR1, etc.) were successfully identified. The protein-protein interaction (PPI) network indicated the important role of STAT3. The functional enrichment of GO and KEGG pathway showed that LXTYF is most likely to influence MAPK and PI3K-Akt signaling pathways for AICH treatment. From the RNA-seq of AICH-rats, 583 differential mRNAs were identified and 14 of them were consistent with the putative targets of LXTYF for AICH treatment. The KEGG pathway enrichment also implied that the MAPK signaling pathway was the most correlated one among all the related signaling pathways. Many important targets with expression changes of LXTYF for AICH treatment and their related pathways are great markers of antioxidation, anti-inflammatory, antiapoptosis, and lowering blood pressure, which indicated that LXTYF may play mutiroles in the mechanisms for AICH treatment.

**Conclusion:**

The LXTYF attenuates AICH partially by antioxidation, anti-inflammatory, and antiapoptosis and lowers blood pressure roles through regulating the targets involved MAPK, calcium, apoptosis, and TNF signaling pathway, which provide notable clues for further experimental validation.

## 1. Introduction

Nowadays, stroke is still a major threat to people's health. Statistics from the World Health Organization show that 15 million people suffer the disease worldwide each year [1]. Less frequently than ischemic strokes, intracerebral hemorrhage (ICH) makes up 10%-20% of all strokes. But a higher fatality rate is caused in the acute phase of intracerebral hemorrhage (AICH), up to 40%-54% [2, 3]. ICH leads to stroke damage when a troubled brain blood vessel bursts, blood leaks, and the brain is loss of oxygen and blood. Deep brain and nerve damage is made due to the dead blood clot cells and released toxins. High blood pressure is the most common cause of ICH [4]. Various forms of surgical decompression are in widespread use to treat this kind of stroke, but the effect remains controversial. Unfortunately, there is still no proven (in phase 3 trials) beneficial medical treatment for AICH [5, 6].

In the last two thousand years, traditional Chinese medicines (TCMs) have been applied in treating various kinds of diseases in China, including stroke [7, 8]. Compared with Western medical treatment for AICH, TCMs have fewer adverse effects and holistic and systematic influence on more pharmacological activities and efficacies. Currently, an important challenge is to discover proven therapeutic medication for AICH. Buyang Huanwu Decoction, Fufang Danshen Injection, Xueshuantong Injection, and Liangxue Tongyu Formula are of the most common use for ICH herbal patent injections [9]. Among them, Liangxue Tongyu Formula (LXTYF) is a good prescription beneficial for ameliorating AICH, created by Chinese Medical Master Zhongying Zhou, who has 70 years of medical experience in China. It comprises six botanical medicinal materials like *Rheum palmatum* L. (Dahuang, DaH), *Rehmannia glutinosa* Libosch. (Dihuang, DiH), *Panax notoginseng* (Burk.) F.H.Chen (Sanqi, SQ), *Paeonia lactiflora* Pall. (Chishao, CS), *Paeonia suffruticosa* Andr (Mudanpi, MDP), and *Acori Tatarinowii* Schott (Shichangpu, SCP) and two animal medicines like *Bubali Cornu* (Shuiniujiao, SNJ), *Pheretima aspergillum* (E. Perrier) (Dilong, DL). This formula was adapted from Xijiao Dihuang Decoction, an ancient TCM classical prescriptions reserved in one of the classic works of ancient Chinese medicine called Essential Recipes for Emergent Use Worth A Thousand Gold, and only replaced *Rhinoceri Asiatici Cornu* (rhinoceros horn) with SNJ for the similar medicinal effects and compliance with animal protection and regulations [10]. According to previous research, LXTYF may improve symptoms of AICH, attenuate nerve injury, and remove blood stasis of patients, as well as ameliorate brain edemas in AICH rats [11, 12]. For Ma's 96 AICH patients and Guo's 168 AICH patients in treatment group, LXTYF combined with Western medicine treatment was reported to have a better therapeutic effect than Western medication only [12, 13]. It attenuated symptoms of AICH, promoted the absorption of intracerebral hematoma, improved the recovery of consciousness, and reduced the nerve damage of patients, thus improving the prognosis and reduced the disability rate [12, 13]. Li's previous work has indicated that LXTYF had great pharmacology efficacy on AICH by in vivo and in vitro experiment [14].

**Figure 1:**
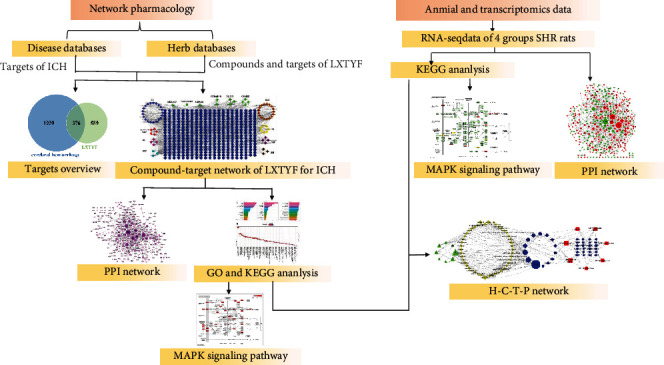
Outline of the study. Network pharmacology method and transcriptomics data were applied to explore the LXTYF effect on AICH.

In our research, network pharmacology method is applied to predict active components, targets, and pathways of LXTYF to alleviate AICH. TCMs provide multiple components, targets, and pathways, also with similar characteristics with systems biology and network pharmacology. This indicates that we can use the approach of the network pharmacology to deeply investigate the mechanisms of TCMs to alleviate AICH [15]. Further, pivotal human genes and pathways were verified after the conversion of rat transcriptomics RNA-seq data (Figure 1). Especially, in the animal experiment, the commonly used AICH model of spontaneous hypertension rats (SHRs) induced by collagenase was made, and LXTYF administration was then conducted for RNA-seq.

This study is aimed at digging into the system pharmacological mechanism of Liangxue Tongyu Formula (LXTYF) on treating intracerebral hemorrhage via network pharmacology method and RNA sequencing data and providing notable clues for further experimental validation.

## 2. Materials and Methods

### 2.1. Herb Formulation Ingredients Collection

The chemical ingredients of six botanical medicinal materials in LXTYF were obtained from Traditional Chinese Medicine Systems Pharmacology Database and Analysis Platform (TCMSP) (http://lsp. http://nwu.edu.cn/tcmsp.php) [16]. TCMSP is a distinctive platform for TCM pharmacology, able to provide herbal information, ADME data for the screening process and related targets of each chemical ingredient. The chemical ingredients of the other two animal medicines in LXTYF were derived from BATMAN-TCM (https://bionet.ncpsb.org/batman-tcm) [17]. BATMAN-TCM is the first online Bioinformatics Analysis Tool for the Molecular mechanism of TCM, which can provide the active ingredients and ranked target prediction of animal medicines for analysis.

### 2.2. Active Ingredients Screening

The active ingredients from herbs of LXTYF were filtered by OB and DL. OB represents the relative amount of a drug absorbed into the blood circulation, and DL is used to describe pharmaceutical properties of compounds [18, 19]. Based on literature and suggestions in TCMSP, OB ≥ 30% and DL ≥ 0.18 were selected as a screening threshold [20–22]. Ingredients that meet the above rules will be preserved for further analysis. Moreover, several active ingredients of Dihuang, Sanqi, and Shichangpu with important pharmacological effects were added into the compound database for analysis based on the literature about chemical composition and the Chinese Pharmacopoeia 2015.

### 2.3. Active Ingredient-Associated Target Prediction

TCMSP and BATMAN-TCM databases are integrated to collect known targets of active ingredients in LXTYF. The target names were obtained from the TCMSP and then converted into Uniprot ID in the “Retrieve/ID Mapping” tool of Uniprot (Universal Protein Resource) database (http://www.uniprot.org/), which is specialized for protein information [23]. To analyze those active components that have unknown target proteins, the following databases were used. PubChem is the world's largest collection of chemical information, in which name, molecular formula, structure, physical properties, and so on can be found [24]. Open Babel toolkit is a toolbox transforming multiple chemical file formats [25, 26]. With the help of these two databases, SMILES formats of those active components were converted. SwissTargetPrediction is a web-based tool used to predict the most possible targets for small molecules. The target predictions are achieved through reverse screening based on the similarity principle [27]. The top 15 potential targets of each active component were predicted from the SwissTargetPrediction database.

### 2.4. ICH-Associated Target Prediction

Different genes linked to ICH were collected from three existing resources with Medical Subject Heading (MeSH) “Cerebral Hemorrhage”. (1) GAD (http://geneticassociationdb.nih.gov/), a public genetic association database serving for human genetic diseases based on integrated datas of more than 5000 published studies [28]. (2) Genecards (https://www.genecards.org/), an automatically mined database of human genes and a very wide range of resources for gene-centric information in the human genome [29, 30]. (3) DisGeNET (http://www.disgenet.org/web/DisGeNET), which integrates data from animal models, GWAS catalogues, expertly curated repositories, and the scientific literature, constructing a comprehensive platform studying human disease-associated genes and variants [31].

### 2.5. Network Construction and Key Nodes Analysis

Putative targets of LXTYF for the treatment of AICH were obtained based on the interaction data between active ingredient-associated targets and ICH-associated targets, and Cytoscape software (Version 3.2.1) was utilized to visualize the network for AICH-associated-active ingredients and targets. Degree and betweenness centrality are important topological parameters of networks and were calculated in Cytoscape to measure the topological importance of target and compound nodes [32–34]. The nodes were identified as key targets and key chemical ingredients according to the “Degree” and “Betweenness Centrality” values, which were larger than the average degree and average betweenness centrality of all nodes in the network.

### 2.6. PPI Network Construction

Proteins are rare to achieve specific functions alone and tend to form macromolecular complexes by interaction in a single cell to complete biological functions [34]. So, it is necessary to analyze the interactions in the proteins-proteins interaction (PPI) network. STRING 11.0 (https://string-db.org/) is a database with integrated data from several active interaction sources such as text mining, experiments, databases, and coexpression. It uploads gene datasets as input and visualizes them in a network for the next analysis [35]. In this study, STRING was used to analyze the interactions between targets (cut − off critical level of high interaction score confidence = 0.95) of LXTYF for AICH.

### 2.7. Gene Ontology and Pathway Enrichment

The ClusterProfiler package installed in R offers the *groupGO* method to classify genes [36]. There are three aspects associated with GO terms as follows: cellular components (CC), molecular functions (MF), and biological processes (BP). Also, by hypergeometric distribution, *enrichKEGG* function is provided to proceed with enrichment test for KEGG pathways. *p.adjust values* are also estimated to prevent a high false discovery rate (FDR). Pathways that the *p*.adjust ≤ 0.05 were reserved to analyze.

### 2.8. Animals and Reagents

Eight crude herbs of LXTYF were purchased from Tongrentang Chinese Medicine Company (Since 1669). Spontaneous hypertension rats (SHRs) were purchased from Beijing Vital River Laboratory Animal Technology Co., Ltd., and animal license number was SCXK (Beijing) 2016-0006. Collagenase type VII was purchased from Sigma-Aldrich (St Louis, MO, USA). Brain stereotaxic instruments and electric drills were bought from Yuyan Instruments Co., Ltd. (Shanghai, China). TJ-1A microinjection pump was purchased from Lange Constant Flow Pump Co., Ltd. (Baoding, China). Microneedle was purchased from Gaoge Industry & Trade Co., Ltd. (Shanghai, China). Chloral hydrate and penicillin were bought from Sinopharm Chemical Reagent Co., Ltd. (Shanghai, China). TRIzol® Reagent and TBS380 Picogreen were purchased from Invitrogen (Carlsbad, CA, USA). Magnetic frame Ribo-Zero Magnetic kit was bought from EpiCentre (USA). TruSeqTM Stranded Total RNA Library Prep Kit, UNG enzyme, HiSeq 4000 PE Cluster Kit, and HiSeq 4000 SBS Kit (300cycles) were purchased from Illumina (San Diego, CA, USA). Certified Low Range Ultra Agarose was purchased from Bio-Rad (Hercules, CA, USA).

### 2.9. LXTYF Preparation and Animal Administration

LXTYF consisted of *Cornu Bubali* (SNJ), *Rheum palmatum* L. (DaH), *Rehmannia glutinosa* Libosch. (DiH), *Panax notoginseng* (Burk.) F.H.Chen (SQ), *Radix paeoniae rubra* (CS), Moutan Cortex (MDP), *Pheretima aspergillum* (DL), and *Acori Tatarinowii Rhizoma* (SCP). The crude herbs mixed in the weight ratio of 30 : 10 : 20 : 5 : 15 : 10 : 10 : 10 and extracted by means of reflux in ethanol and water for 3 hours. LXTYF decoction was prepared with 8 : 1 water/oil.

All procedures concerning animals in this study were approved by the ethics committee of Nanjing University of Chinese Medicine. Spontaneous hypertension rats (SHRs) were randomly assigned into cages and adapted for experiment environment in the SPF-room for one week, with food and water regularly and temperature at 20-25°C. 20 male SHR rats (230-260 g) were divided into four groups randomly for modeling and experiments: control group (CG), control group with LXTYF treatment (CGT), ICH-model group (MG), and AICH model with LXTYF-treatment group (MGT), with 5 SHR rats in each group. Rats in CG and MG were given physiological saline, while CGT and MGT rats received intragastric administration of LXTYF decoction for 5 days. 12 h-fasting-rats in MG and MGT were made AICH models by using 0.12 U collagenase of 2 *μ*l and brain stereotaxic instrument. According to our previous study, the middle dose of 11.55 g/kg of LXTYF has the best efficiency on survival time and rate for rats with AICH. So, all the dosage of the following administration was 11.55 g/kg. After 24 hours of modeling, rats were sacrificed by decapitation, and brain tissue of the rat was quickly obtained from where the microinjector was inserted. The specimens of brain tissues were frozen in liquid nitrogen and stored in -80°C refrigerator for RNA-seq.

### 2.10. Sequencing for Differentially Expressed mRNA

The specimens of brain tissues were obtained from the rats (4 groups with 3 replicates). Total RNA was extracted from brain tissue specimens and tested for its concentration and purity. 5 *μ*g of rat total RNA was started, and the concentration was ≥250 ng/ml. OD260/280 was between 1.8 and 2.2. After removing the rRNA, indexed libraries were prepared from purified RNA with TruSeqTM Stranded Total RNA Library Prep Kit (Illumina) according to the manufacturer's instructions. Randomly interrupted with metal ions, 2000 bp mRNA fragments were obtained. The first strand cDNA synthesis was performed according to the mRNA fragments as described in the Illumina kit. The second strand cDNA synthesis was modified by exchanging dTTP with dUTP. After adding end repair mix to double-stranded cDNA, blunt ends were performed, and A bases were added to attach the Y-shaped adapters. The UNG enzyme digested the second-strand cDNA, and the library only contained the first-strand cDNA. Bridge PCR amplification was performed on cBot, and 2∗150 bp sequencing was performed on Illumina Hiseq. Further details can be found in the references [37, 38].

### 2.11. Verification of Related mRNAs and Pathways

With the thresholds of ≥2-fold and the *p* value of ≤0.05 via edgeR (the Empirical Analysis of Digital Gene Expression in R, v3.2.4) software, differentially expressed mRNAs were identified especially the MGT and MG. Rat genes were converted into human genes based on homology mapping through the Biomart package in R. Compared with the putative targets that LXTYF treats for AICH, PPI network of verified genes and other genes was constructed in STRING and Cytoscape software.

KEGG orthology-based annotation system (KOBAS) is a web server for annotation and identification of enriched pathways and diseases [39]. In our research, enrichment analysis of the differentially expressed mRNAs was performed using KOBAS 3.0 (http://kobas.cbi.pku.edu.cn/anno_iden.php). In the KEGG database, Fisher's exact test was used as a statistical method, and Benjamini and Hochberg was used as an FDR correction method. Enriched pathways that *p* value≦0.05 were identified compared with former pathway enrichment prediction.

### 2.12. Herb-Compound-Target-Pathway(H-C-T-P) Network Construction

Compared the pathways enriched by the network pharmacology method with those enriched by RNA-seq data, the common and most likely pathways are obtained. Differentially expressed genes related to common pathways are achieved from the RNA-seq data. The verified gene-related compounds and herbs are found from the collected network pharmacology datas, connections among them were shown in the Herb-Compound-Target-Pathway(H-C-T-P) network and visualized in Cytoscape software.

## 3. Results

### 3.1. Active Ingredients of LXTYF

A total of 517 compounds of 8 herbs in LXTYF were collected from TCMSP and BATMAN-TCM database after removing duplicate items, including 119 compounds from CS, 92 compounds from DaH, 76 compounds from DiH, 119 compounds from SQ, 55 compounds from MDP, 105 compounds from SCP, 6 compounds from SNJ, and 8 compounds from DL (S1 Table).

Considering the drug formation of the compound and metabolism after being absorbed into the human body, 81 active ingredients of LXTYF filtered by OB and DL were screened out from the 517 compounds with ADME parameters as shown in Table 1. Specifically, there were 30 active ingredients in CS, 19 active ingredients in DaH, 12 active ingredients in MDP, 12 active ingredients in SQ, 7 active ingredients in DL, 6 active ingredients in SNJ, 5 active ingredients in DiH, and 5 active ingredients in SCP. Among them, beta-sitosterol, sitosterol, and stigmasterol are shared by three herbs. Quercetin, kaempferol, (+)-catechin, paeonol, paeoniflorin_qt, benzoyl paeoniflorin, 4-guanidino-1-butanol, cholesterol, and guanidine are common active ingredients of two herbs.

### 3.2. Putative Targets of LXTYF for the Treatment of AICH and Network Construction

**Figure 2:**
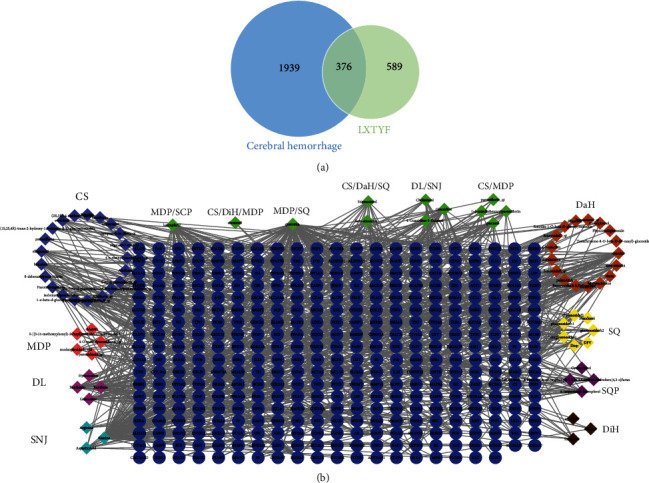
Network construction. (a) Veen diagram describing targets distribution of LXTYF and ICH. (b) The network of Putative Component-Target. Blue ellipses refer to 376 targets of LXTYF for AICH treatment. Different color triangles represent 76 active components in LXTYF. In particular, green triangles stand for common compounds of three and two herbs.

On the basis of the 81 active ingredients of LXTYF above, 965 targets were collected from two databases with no duplicate values. Among them, 874 targets of the active ingredients were obtained from the TCMSP (S2 Table), and 149 targets of those active components that have unknown target protein were predicted from the SwissTargetPrediction database (S3 Table). From the GAD, Genecards, and DisGeNET database, 2315 targets relating to ICH were collected (S4 Table). After overlapping the 2315 targets with former 965 targets, 376 targets of LXTYF for treating AICH were obtained as shown in Figure 2(a). At the help of Cytoscape, a putative component-target network composed of 452 nodes and 890 edges were constructed based on 376 targets and the corresponding 76 components as Figure 2(b).

### 3.3. Network Analysis and Key Nodes Screening

Topological analysis is an effective approach to evaluate the above-mentioned component-target network based on the degree and betweenness centrality parameters (S5 Table for details). The following are the obtained results of the network topological analysis: network heterogeneity (2.144), network density (0.009), and shortest paths (202052, 99%). In the component-target network, the first three targets are PTGS2 (degree = 24), PTGS1 (degree = 21), and ESR1 (degree = 17). The first three components are quercetin (degree = 112), alanine (degree = 104), and kaempferol (degree = 39), which are, respectively, from MDP/SQ, SNJ, and MDP/SCP.

From an in-depth analysis of the complete network above, key components and targets with imporant biological functions can be identified. The nodes (compound and target) whose degrees and betweeness centralities are both larger than the average ones were identified as key targets and chemical ingredients. The average degree of the nodes is 3.938, and 122 nodes are larger than this number. The average betweenness centrality of nodes is 0.006549 and 85 nodes are larger than it. In general, 74 key nodes (35 key components from 76 components and 39 key targets from 376 targets) were obtained, which was shown in Table 2. They were extracted and the connections of the key-compound-target network were displayed in S1 Fig. The first 3 key targets are PTGS2, PTGS1, and AR, and the first 3 key components are quercetin, kaempferol, and emodin, which may all have a great effect on AICH treatment.

### 3.4. PPI Network Construction

**Figure 3:**
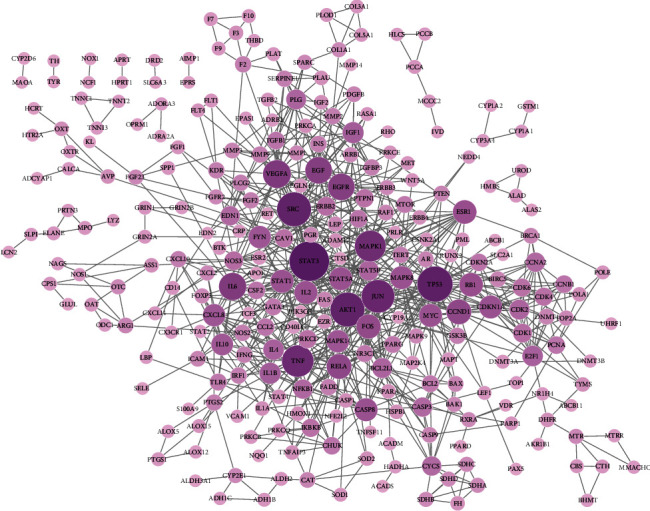
PPI network of LXTYF targets for treating intracerebral hemorrhage (larger degree of the target node with larger and deeper color).

For analysis of the proteins-proteins interaction network of LXTYF for AICH, STRING 11.0 was used to analyze the targets of LXTYF (Results in S6 Table). Apart from the targets independent of the network and the confidence level of interaction score less than 0.95, 275 related targets (from 376 targets) and 850 interactions were included in the network visualized by Cytoscape in Figure 3. The importance of key proteins was then evaluated in light of the degree of the nodes exported from the STRING database. It is clear that the STAT3 (degree = 45) is much larger than other nodes in the network (Avg.numbers of neighbors = 6.182), followed by TP53, AKT1, SRC, JUN, TNF, and MAPK1 (degree values larger than 30). The topological parameters of the PPI network are shown in S7 Table.

### 3.5. Gene Ontology and Pathway Enrichment for Putative Targets

**Figure 4:**
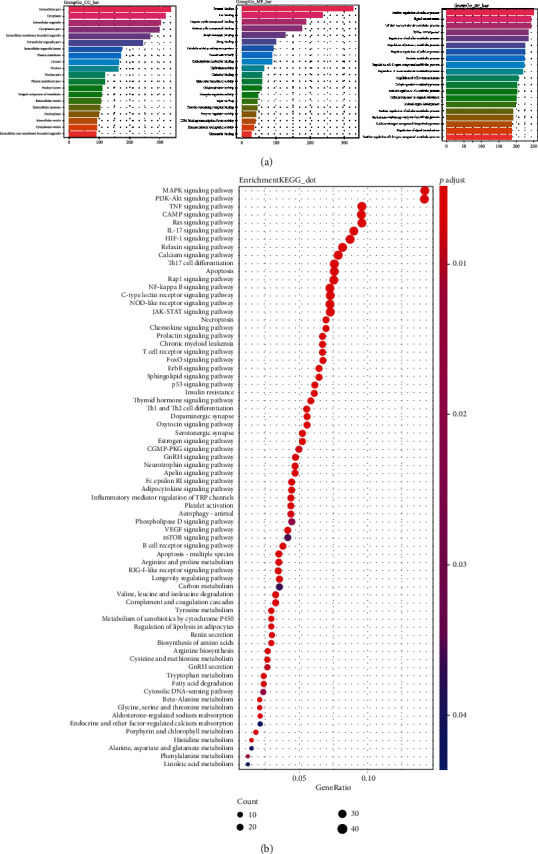
Enrichment analysis. (a) *groupGO* function for go ontology of 376 targets. The horizontal axis describes the number of genes and the vertical axis describes the GO terms. (b) *enrichKEGG* function for KEGG pathway enrichment analysis of 345 targets. The horizontal axis denotes generatio, and the vertical axis represents KEGG pathway terms. The size of the spot indicates the gene numbers enriched in each pathway and color indicates FDR value.

For further study about the putative targets of LXTYF on AICH action, Clusterprofiler package in R was used for analysis due to its higher classification and enrichment accuracy, as well as the visualization module for the results [36]. 376 targets above were applied to run the package for gene classification and KEGG enrichment analysis (FDR ≤ 0.05).  Figure 4(a) depicted the top 20 GO terms of biological processes (BP), molecular functions (MF), and cellular components (CC), respectively. Results covered that 376 targets of LXTYF for treatment on AICH in BP were mainly associated with positive regulation of the cellular process, signal transduction, and cellular macromolecule metabolic process. In the aspect of MF, the targets mainly participated in the process of protein binding, ion binding, and cyclic compound binding. Obviously, the cellular components mainly occur in the cytoplasm, intracellular organelle, and nucleus.

**Figure 5:**
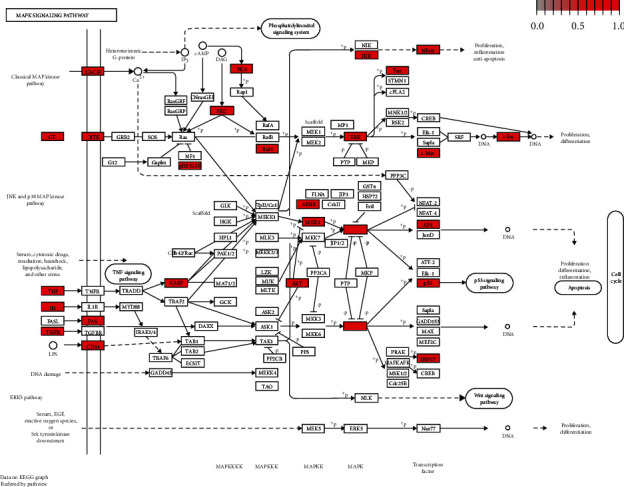
MAPK signaling pathways related to LXTYF for AICH. KEGG ID=hsa04010, enriched with 49 genes.

Figure 4(b) showed 72 enriched KEGG pathways identified using the Clusterprofiler package. The top 5 enriched pathways were MAPK signaling pathway, PI3K-Akt signaling pathway, TNF signaling pathway, cAMP signaling pathway, and Ras signaling pathway, with 49,49,33,33, and 33 genes, respectively. See S8 Table for results of putative targets for KEGG pathway enrichment analysis. The most highly enriched pathways related to LXTYF for AICH were the MAPK signaling pathway and the PI3K-Akt signaling pathway. MAPK signaling pathway was shown in Figure 5.

### 3.6. Identification of Mutually Expressed mRNAs

**Figure 6:**
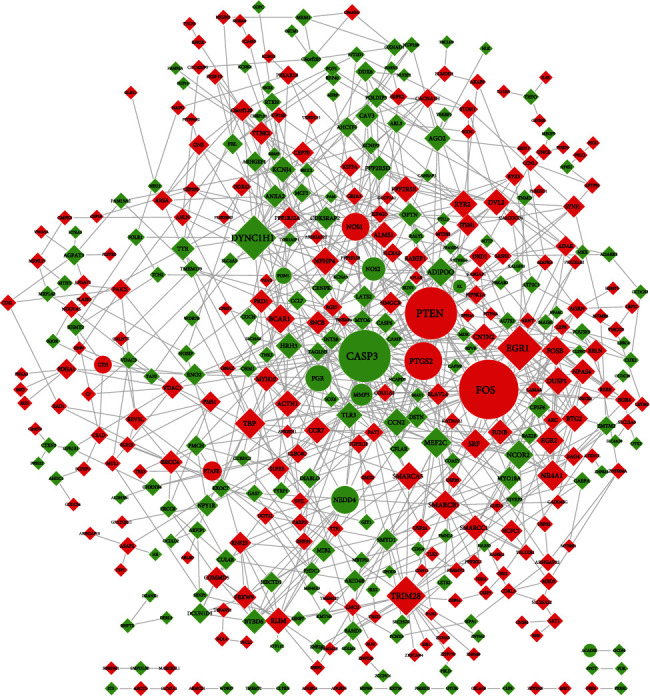
PPI network of identified genes and other differentially expressed genes from the RNA-seq. Compared with the MG, green shapes represent downregulated mRNAs, and red shapes represent upregulated mRNAs in MGT. Ellipses represent the verified genes treated by LXTYF for AICH, and diamonds represent other genes differentially expressed. A larger degree of the target nodes with larger shapes.

With a fold-change cutoff of 2 (*p* ≤ 0.05), the analysis of differently expressed genes among four groups from the RNA-seq data was shown in Table 3 (3 duplicate values in each group). 766 differentially expressed genes between MG (AICH group) and CG (Control group) showed AICH-induced pathological changes in the SHR rats. Also, 583 differentially expressed mRNAs were identified between MGT and MG, with 308 upregulated mRNAs and 275 downregulated mRNAs. Detailed information for genes is displayed in S9 Table. The 583 rat genes were converted into human genes based on homology mapping using Biomart package in R. Removing the duplicate values, 519 human genes were obtained. Compared with the putative 376 targets that LXTYF for AICH, 14 genes were verified as follows: PTEN, CTH, PTGS2, PTAFR, FOS, NOS1, NOS2, PGR, MMP3, PON1, NEDD4, ACADSB, KL, and CASP3. The interaction network between 14 genes and other 403 genes was shown in Figure 6 at the help of the STRING database (cut off of confidence = 0.4) and Cytoscape. Obviously, FOS, CASP3, and PTEN played an important role among them. Many important targets with expression changes, especially the 14 verified genes and the important gene nodes in the PPI network, are great markers of antioxidation, anti-inflammatory, antiapoptosis, and lowering blood pressure, which indicated that LXTYF may play mutiroles in the mechanisms for AICH treatment.

### 3.7. KEGG Pathway Analysis of the mRNAs

In total, 519 differentially expressed mRNAs were submitted to KOBAS. The 33 enriched pathways that *p* value ≤ 0.05 were shown in Table 4. Expression changes of the genes and their related pathways of LXTYF for AICH treatment, such as the MAPK signaling pathway, calcium signaling pathway, Apoptosis, TNF signaling pathway, cGMP-PKG signaling pathway, cAMP signaling pathway, and HIF-1 signaling pathway, are involved in the biological processes of the antioxidation, anti-inflammatory, antiapoptosis, and lowering blood pressure.

**Figure 7:**
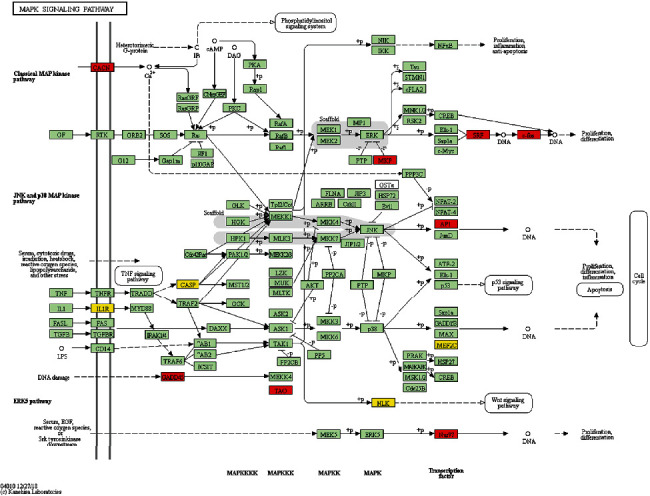
The most correlated MAPK signaling pathway enriched in KEGG mapper. All green boxes represent targets of MAPK signaling pathway in human. 8 red boxes (GADD45(GADD45G), MKP (DUSP1), c-fos (FOS), AP1 (FOS), Nur77(NR4A11), SRF, CACN (CACNA1H), and TAO(TAOK2)) represent upregulated targets. 4 yellow boxes (IL1R(IL1RAP), MEF2C, NLK, and CASP(CASP3)) represent downregulated targets.

Obviously, most pathways are metabolic pathways, and the analysis identified MAPK signaling pathway as the most correlated signaling pathway, which is consistent with the prediction of the former related pathways of LXTYF intervention on AICH. At the help of the KEGG mapper function in the KEGG database, 11 genes were enriched in the MAPK signaling pathway in Figure 7 as follows: GADD45G, DUSP1, FOS, NR4A11, IL1RAP, MEF2C, NLK, SRF, CASP3, CACNA1H, and TAOK2.

### 3.8. Herb-Compound-Target-Pathway(H-C-T-P) Network Construction

**Figure 8:**
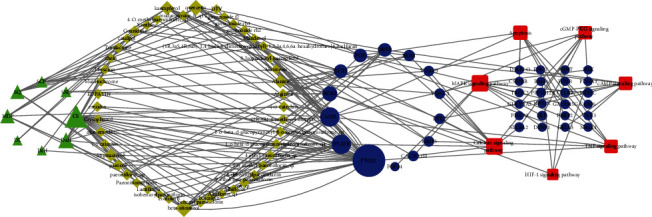
Herb-Compound-Target-Pathway(H-C-T-P) verified network related to the treatment of LXTYF for AICH in RNA-seq. Green triangles represent 8 herbs, yellow diamonds represent 46 compounds, blue ellipses represent 38 targets, and red round rectangles represent 7 pathways. A larger degree of the target nodes with larger shapes.

In view of the mapping results between putative 72 pathways enriched by network pharmacology and 33 pathways and genes enriched in RNA-seq, 7 most common possible pathways were obtained. 38 differently expressed genes related to 7 pathways were achieved in RNA-seq data. Relevant 46 compounds and 8 herbs of the verified 14 genes were achieved from the data collected by network pharmacology. Network connections of the Herb-Compound-Target-Pathway (H-C-T-P) verified in RNA-seq were shown in Figure 8 and Table S10. In this network, nodes of degree among 10~33 which have great topological importance were as follows: CS and DaH in Herb, beta-sitosterol and quercetin in compound, PTAFR and CASP3 in Target, MAPK signaling pathway, and calcium signaling pathway in pathway. Details of connections among the 8 important nodes were shown in Table 5.

The results visually clarify the mechanisms of the multicomponent, multitarget, and multipathway of LXTYF on the treatment of AICH. Further studies can be made and deeply investigate mechanisms upon the connections among the H-C-T-P network of LXTYF to treat AICH in our study.

## 4. Discussion

TCM has been developed and applied in China for more than 2000 years to prevent and treat diseases. To explore active ingredients, predict the targets, and reveal compounds action and drug-gene-disease associations in depth, TCM network pharmacology approach was constructed through “network target, multicomponents” mode [40]. The method is of predictable and systematic features. However, systematically investigating the scientific foundation of TCM formula for diseases at the molecular level is still a great challenge [40]. On the other hand, the development of modern molecular biology and genomic technologies, together with more advances in omics data and computational methods, can help to investigate complex biological pathways and mechanisms of medicines from molecular and cellular levels [41]. Li's previous study in our group showed LXTYF inhibited apoptosis and promoted proliferation in PC12 cells damaged by L-glutamate. Also, a decrease in levels of functional proteins in SHRs such as TNF-a, IL-1, D2D, S-100B, and NSE after LXTYF administration showed LXTYF exerted great pharmacology effect related to anti-inflammatory, anticoagulation, and blood vessel protection activity [14]. Huang's study also showed the cooling-blood and activating-blood effects of LXTYF and its parts via decreases in rectal temperature, blood viscosity, and plasma viscosity of SD rats [42]. In a rat model induced by intrastriatal autologous blood injection, LXTYF significantly ameliorated brain edema after AICH by upregulating metalloproteinase-1 (TIMP-1) and inhibiting metalloproteinase-9 (MMP-9) expression [11].

Network pharmacology and transcriptomics data were applied in this study to understand the underlying different mechanisms and actions of LXTYF for AICH treatment. We successfully identified 76 active ingredients (quercetin, alanine, kaempferol, and so on) of LXTYF and 376 putative targets to alleviate AICH (PTGS2, PTGS1, ESR1, and so on). PPI network indicated the important role of STAT3 in the proteins-proteins interaction. Gene Ontology and KEGG pathway enrichment analysis indicated that LXTYF is most likely to influence the MAPK signaling pathway and PI3K-Akt signaling pathway. From the RNA-seq data of AICH-rats, 583 differentially expressed mRNAs were identified, and 14 of them were consistent with the putative targets of LXTYF treatment of AICH. KEGG pathway enrichment results showed that the MAPK signaling pathway was the most correlated one among the pathways.

### 4.1. Ingredients and Targets of LXTYF for AICH

Ingredient-target-pathway analysis on herb prescriptions is helpful to uncover the complicated mechanisms underlying diseases and the therapy. Network pharmacology is an effective method to deeply investigate the mechanisms of TCMs to treat AICH through muti-ingredients and mutitargets. In this study, 76 active ingredients and 376 targets of LXTYF for treating AICH were obtained at the help of 2315 ICH targets, the deeper results than the 34 active ingredients, 146 targets, and the 1436 ICH targets in our former study [14].

The active ingredients involved in eight herbs were screened by OB and DL values, which represent the vast majority of the ingredients absorbed into the blood circulation and pharmaceutical properties of compounds in the prescription, and form a relatively complete chemical component library. To some extent, they can reflect the pharmacodynamic ingredients of LXTYF that may play a role in the clinical medication. Results of the active component-target network of LXTYF to treat AICH showed that most components affected multiple targets. Quercetin, alanine, and kaempferol from MDP/SQ, SNJ, and MDP/SCP acted on 112, 104, and 39 targets, respectively. They are of high OB and DL values and regulated most targets for AICH treatment, also with anti-inflammatory, antioxidant, and antiapoptotic properties. The findings were consistent with previous studies as follows. Alanine was reported to have antioxidant and procoagulant efficacy [10, 43]. D-alanine D-leucine enkephalin (DADLE) has anti-inflammatory, antioxidant, neuroregenerative, and antiapoptotic properties [44]. Quercetin can promote neuronal and behavioral recovery by its antioxidant activity and restraining inflammatory response and apoptosis in a rat model of ICH [45, 46]. Kaempferol can effectively prevent nitrosative-oxidative stress after ischemia/reperfusion via the mechanism of inhibiting the nitrotyrosines and preventing apoptosis by degrading caspase-9 activity and poly-(ADP-ribose) polymerase in model rats [47]. On the other hand, kaempferol glycosides suppress brain injury and inflammation by inhibiting the activation of NF-*κ*B and STAT3 in stroke rats [48]. So, quercetin, alanine, and kaempferol might reasonably be regarded as crucial active compounds of LXTYF to treat AICH.

Numerous constituents make up the complexed TCM system, and combinations of them play a role in the treatment of diseases. From the H-C-T-P network, the compounds of animal herbs like xanthine and guanidine in DL and guanidine, arginine and alanine in SNJ play vital role in the putative mechanisms of LXTYF to treat AICH. SNJ has been a good substitute for *Rhinoceri Asiatici Cornu* (a highly endangered species) since the 1970s because it is abundant, has low price and with similar inorganic, amino acid constituents, and comparable pharmacological properties [10]. Thus, it is favorable to keep the balance between legislation and apparent indispensability of *Rhinoceri Asiatici Cornu* in TCM. The research and discovery of the active ingredients in this study will contribute to find alternative medicines of endangered animals to better protect animals and treat diseases.

Via evaluating topological paramters, among all the putative targets of LXTYF associated with AICH, PTGS2, Bax, TNF, JUN, IL1B, and NOS2 are examples of relatively important targets which are involved in MAPK, PI3K-Akt, TNF, and apoptosis signaling pathways, while RNA-seq analysis showed that the LXTYF administration significantly regulated 583 gene transcriptions in AICH rat models. Combined with putative targets in network pharmacology, a total of 14 genes were verified. PTEN, CTH, PTGS2, PTAFR, FOS, and NOS1 are six upregulated genes, and NOS2, PGR, MMP3, PON1, NEDD4, ACADSB, KL, and CASP3 are eight downregulated genes. Especially CTH, PTAFR, PGR, PON1, NEDD4, ACADSB, and KL are 7 new genes compared with our previous study [49]. And PPI network of them showed that FOS, PTEN, and CASP3 were core genes playing important roles among the differently expressed genes.

PTGS2 and PTGS1 (Prostaglandin G/H synthase, PTGS) are genes that code for the COX-2 and COX-1(COX enzymes, cyclooxygenase) proteins, respectively [50]. COX enzyme is a key therapeutic target, and its inhibitors such as NSAIDs are widely used in clinical treatment for inflammation, pain, fever, and son on [50]. But inhibition of COX-2 conveys a definite risk of myocardial infraction and stroke due to thrombosis and hypertensive effects [51]. Therefore, upregulation of the PTGS2 gene indicates that LXTYF may reduce the risk of blood pressure elevation, which is conducive to antihypertensive therapy in the early stage of an intracerebral hemorrhage.

Phosphatase and tensin homolog deleted on chromosome 10 (PTEN) is a tumor suppressor, a proapoptotic gene mainly via inactivation of PI3K/AKT signaling pathway, and *PTEN* conditional knockouts have become a potentially approach targeting cancer and obesity treatment [52]. But in stroke treatment, PTEN is a double-edged sword [53]. The previous study showed that PTEN was upregulated in AICH stage, and its inhibition has been reported to be neuroprotective against ischemic stroke in experimental models [53, 54]. While it has been demonstrated that PTEN deletion can lead to cognitive impairment and PTEN loss in neurons and astrocytes is, respectively, unfavorable to long-term functional recovery, exacerbates damage, enhances astrogliosis, and induces inflammation [53]. So, upregulation of PTEN in our study may show that LXTYF treatment has no harm on cognition function and play roles on neuronal-damage recovery after AICH.

Nitric oxide synthases (NOS), with 3 isoforms called neuronal NOS (nNOS/NOS1), inducible NOS (iNOS/NOS2), and endothelial NOS (eNOS/NOS3), form NO and play dual roles of damage and protection in ICH [55]. NO formed via NOS1 and NOS3 gives rises to vasodilatation, hypotension, inhibitions of platelet aggregation, and adhesion, and as an antioxidant, it exerts other beneficial actions. NOS1 has great significance in controlling salt-water balance, and blood pressure, especially the long-term blockade of NOS1 by 7-NI, a specific inhibitor of NOS, leads to hypertension in rats [55], while NO through NOS2 during inflammation increases oxidative and nitrative stress, causing neurodegeneration and enhancement of apoptosis [56]. Selective NOS2 inhibitors that exhibit neuroprotective properties can be candidate treatments for acute ischemic stroke [57]. So, LXTYF may play function of vasodilatation and antihypertension via upregulation of NOS1 and supress oxidative stress and apoptosis via downregulation of NOS2 to treat AICH.

FOS is one of the components of AP1, the inducer of cell proliferation. It can activate nerve growth factor and has gene repair activities after neuronal death in the stroke model. Knockdown of c-fos enhances necrosis significantly [58]. But serving as an immediate-early gene, its role of expression is complex and has neurotoxic and neuroprotective effects. Especially its neuroprotective effect is mainly on gene repair activities after neuronal death, with intact c-fos transcript playing roles in the necrosis core and oxidative DNA lesions [58]. So, upregulated changes of FOS in RNA-seq showed that LXTYF may have neuroprotective effect and attenuated oxidative DNA lesions and necrosis after AICH, similar to 7-nitroindazole (7-NI). 7-NI is a specific inhibitor of NOS and can increasec-fos mRNA levels and attenuate oxidative DNA lesions, nitric oxide, and necrosis after stroke [58]. But several previous reports showed that upregulation of c-Fos after AICH leads to neuronal apoptosis, and FOS is regarded to be neurotoxic. So, role of FOS in AICH also needs further study.

Caspase 3, a member of caspases family involved in cell death, has been recognized as a crucial mediator of neuronal apoptosis [59]. Stroke is usually of great neuronal cell death and serum caspase-3 concentrations rise in rat models and AICH patients [59–61]. Downregulation of Caspase 3 after administration in this study shows that LXTYF may treat AICH via apoptosis inhibition.

### 4.2. Pathway Analysis of LXTYF for AICH Treatment

Multicomponent, multitarget, and multipathway are general features of TCMs, and LXTYF has therapeutic effects by making contributions to several pathways. In this present work, putative targets were enriched significantly in 72 KEGG pathways, and the two most important enriched pathways were the MAPK signaling pathway and PI3K-Akt signaling pathway. Our previous study has verified that LXTYF inhibited the apoptosis to treat AICH based on the PI3K-AKT pathway on the level of transcription and protein [14]. Meanwhile, MAPK (mitogen-activated protein kinase) signaling pathway is the most important enriched and verified pathway in our RNA-seq analysis, which plays roles in modulating oxidative stress, inflammatory action, and cell death [62]. It can response to extracellular stimuli, then sequential phosphorylation of kinase cascades MAPKK kinases (MAPKKKs), MAPK kinases (MAPKKs), dual phosphorylation of the tripeptide motif (Thr-X-Tyr), and MAPK were activated, thus transmitting signals to the nucleus [63]. MAPK family consists of four subfamilies: extracellular signal-regulated kinase 1/2 (ERK1/2), p38 kinase, c-Jun amino terminal kinase (JNK), and ERK5. It has been viewed as a potential therapeutic target for stroke and hemorrhagic cerebral vascular disease [64]. Studies have found that the balance of MAPK activities determines cell fate. ERK is activated by various growth factors and shows benefits to cell growth and cellular survival. In contrast, p38 and JNK are activated by stress conditions, leading to inflammation and apoptosis via various mechanisms [65]. Some preclinical researches proved that MAPK inhibition (MAPKi) can dramatically increase the efficacy of immunotherapy, especially in cancer types [66]. Proinflammatory cytokines (interleukin-1beta (IL-1*β*), tumor necrosis factor-alpha (TNF-*α*)) and transforming growth factor-beta (TGF-*β*) activate p38 MAPK, and its specific inhibitors were used as the treatment of peripheral inflammatory diseases [64]. It is known that p38s and ERK5 can trigger phosphorylation of MEF2c and then increase c-Jun expression [67, 68], which is distinct among several diseases like brain ischemia, injury, cell death, neuro-inflammatory, and neurodegenerative disease [69]. MEF2C, a member of the myocyte-enhancer factor 2 (MEF2) group of transcription factors, was verfied to be downregulated. Thus, it is reasonable to speculate that p38 MAPK was inhibited; phosphorylation of MEF2C and c-jun mRNA transcription was decreased to prevent inflammatory response and apoptosis for treatment of AICH after LXTYF administration [70, 71]. Proteins of the Fos and Jun families can form homodimers or heterodimers called AP-1 (activating protein-1) to mediate transcriptional activation and DNA binding activity on target genes, involved in apoptotic functions and cell proliferation [66, 72]. Most time c-Fos is a necessary inducer of cell proliferation during the cell cycle phases in growing cells [67]. FOS and FOSB, verified to upregulated, are possible to play the proliferative role and induce compensatory cell proliferation via JNK and ERK pathways for the treatment of AICH after LXTYF administration. Dual-specificity protein phosphatase (DUSP) is an antiapoptotic phosphatase, which can dephosphorylate and inactivates the downstream effectors such as JNK, p38, and ERK [65, 73]. Downregulation of DUSP1 and increase of JNK phosphorylation were observed in the IR injury of the heart. Reproduction of DUSP1 can not only reduce the cellular death and shows the protective effects but also limit inflammatory responses upon LPS stimulation [65, 73, 74]. So, upregulation of DUSP1 shows that LXTYF may have a decrease in MAPKs phosphorylation and inactivates JNK and p38 pathways, suppressing inflammation and apoptosis to treat AICH.

Moreover, the other pathways such as the calcium signaling pathway, apoptosis, TNF signaling pathway, cGMP-PKG signaling pathway, cAMP signaling pathway, and HIF-1 signaling pathway are the common signaling pathways of differentially expressed genes and putative targets in LXTYF. The calcium signaling pathway generates Ca^2+^ signals to regulate physiological processes [75]. Research progress shows that low blood calcium leads to increased hematoma volume and poor prognosis of AICH, and calcium intake in the diet can lower the risk of stroke by about 24% [76]. Blood calcium on reducing stroke is mainly from several mechanisms such as inhibition of fat synthesis, lowering of blood pressure, and stability of blood glucose [76]. Activation of the apoptosis pathway is known to contribute to neuronal loss and become the core problem in the pathogenesis of stroke [77]. Bcl-2 family proteins and caspase proteins regulate neuronal apoptosis via mitochondria and ER. What is more, oxidative and nitrosative stress after stroke injury will lead to apoptotic cell death [77]. DIABLO (direct IAP-binding protein with low pI) is released from mitochondria into the cytosol, interacts with multiple IAPs, and removes IAP-mediated inhibition of caspases [78]. So, when it comes to the downregulation of DIABLO, Caspase-3, and Caspase-6 in RNA-seq data, it is reasonable to speculate the mechanism that LXTYF can help to decrease mRNA expression of DIABLO, then reduced inhibition of IAPs and promoted inhibition of caspase-3 and caspase-6. The TNF signaling pathway plays pleiotropic roles in the CNS [79]. Tumor necrosis factors (TNF) consists of soluble TNF (solTNF) and transmembrane TNF (tmTNF), which has a preference for TNF receptor 1 (TNFR1) and TNF receptor 2 (TNFR2), respectively [79]. Observations have suggested that in acute stroke and reduce injury, solTNF action can be inhibited by intraventricular infusion of the TNFR1 decoy receptor, but in the aspect of hippocampal repair and neurogenesis after ischemic injury, tmTNF action through TNFR2 is crucial [79]. Therefore, the adverse effects of anti-TNF therapies in the CNS may be reduced by selectively supressing TNFR1-mediated signaling while sparing TNFR2 activation [79]. BAG4/SODD is an antiapoptotic protein and identified as a silencer of death domain that can bind to TNFR1. High levels of SODD/BAG-4 protect against TNFa-induced cell death. So, elevation of BAG-4 indicates the control of LXTYF on the TNF signaling pathway.

### 4.3. Limitations of the Research

Compared to the previous two papers published in Frontiers in Pharmacology and Journal of Ethnopharmacology, all studies used reasonable models and reliable validation data to detect the network effect of LXTYF to treat AICH and verified some components, targets, and pathways [14, 78]. But our current work is different in the following aspects. We think that comprehensive ingredients in the prescription, the method with low false positive for pathway enrichment, and detailed differential mRNA expression can more systematically and efficiently validate the network pharmacology effects. Results in our study can supplement the mechanisms of LXTYF on AICH in a more systematic and comprehensive way. We collected compounds of all 8 herbs; more ICH targets and PPI network were analyzed in detail. A more structured and detailed network pharmacology approach via packages in R was also applied in our study. Also, compared with the predictive network and RNA-seq analysis results, new genes and new pathways (14 genes and MAPK signaling pathway) were verified and enriched. In the future, more targets and pathways will be explored deeply, conformed, and fully depict the pharmacology mechanism of TCM on AICH.

There are still some limitations to our study. The insufficient research on medicine composition of this prescription, especially the animal herbs, cannot completely dig into all the effective components. Further progress of chemical component separation technology is in great need to fully clarify all chemical components in the prescription. Also, results of PPI network prediction showed that STAT3 may play an important role of the bridge to connect other proteins among the pharmacological actions, but it did not verify in RNA-seq and needs to investigate in the next future. More subsequent experiments on differently expressed mRNA and enriched pathways are necessary to be investigated deeply. Moreover, it is common to see that some genes have dual effects and play opposite roles in different physical conditions, especially some are unsure and need further study. Critical insight for targets and pathways are necessary for mutual promotion between pharmacological research and physiological/pathology research.

## 5. Conclusion

Taken together, network pharmacology method and enrichment analysis in R were applied in our study to identify the 76 active components and 376 putative targets of LXTYF alleviating AICH. Compared with RNA-seq results of AICH rat models and enrichment analysis, we found that LXTYF attenuates AICH partially by regulating the targets involved MAPK, calcium, apoptosis, and TNF signaling pathway and plays antioxidation, anti-inflammatory, antiapoptosis and lowers blood pressure roles in the treatment mechanisms.

## Figures and Tables

**Table 1:** Description of ADME parameters of LXTYF active ingredients.

Herb	Mol ID	Molecule name	MW	OB (%)	DL
CS	MOL001002	Ellagic acid	302.2	43.06	0.43
CS	MOL001918	Paeoniflorgenone	318.35	87.59	0.37
CS	MOL001924	Paeoniflorin	480.51	53.87	0.79
CS	MOL002714	Baicalein	270.25	33.52	0.21
CS	MOL002776	Baicalin	446.39	40.12	0.75
CS	MOL002883	Ethyl oleate (NF)	310.58	32.4	0.19
CS	MOL004355	Spinasterol	412.77	42.98	0.76
CS	MOL005043	Campest-5-en-3beta-ol	400.76	37.58	0.71
CS	MOL006992	(2R,3R)-4-methoxyl-distylin	318.3	59.98	0.3
CS	MOL006999	Stigmast-7-en-3-ol	414.79	37.42	0.75
CS	MOL001921	Lactiflorin	462.49	49.12	0.8
CS	MOL006990	(1S,2S,4R)-trans-2-hydroxy-1,8-cineole-B-D-glucopyranoside	332.44	30.25	0.27
CS	MOL006994	1-O-beta-d-glucopyranosyl-8-o-benzoylpaeonisuffrone_qt	302.35	36.01	0.3
CS	MOL006996	1-O-beta-d-glucopyranosylpaeonisuffrone_qt	332.38	65.08	0.35
CS	MOL007004	Albiflorin	480.51	30.25	0.77
CS	MOL007005	Albiflorin_qt	318.35	48.7	0.33
CS	MOL007008	4-Ethyl-paeoniflorin_qt	332.38	56.87	0.44
CS	MOL007012	4-O-methyl-paeoniflorin_qt	332.38	56.7	0.43
CS	MOL007014	8-Debenzoylpaeonidanin	390.43	31.74	0.45
CS	MOL007016	Paeoniflorigenone	318.35	65.33	0.37
CS	MOL007018	9-Ethyl-neo-paeoniaflorin A_qt	334.4	64.42	0.3
CS	MOL007022	Evofolin B	318.35	64.74	0.22
CS	MOL007025	Isobenzoylpaeoniflorin	584.62	31.14	0.54
DaH	MOL000096	(-)-Catechin	290.29	49.68	0.24
DaH	MOL000471	Aloe-emodin	270.25	83.38	0.24
DaH	MOL000554	Gallic acid-3-O-(6′-O-galloyl)-glucoside	484.4	30.25	0.67
DaH	MOL002235	EUPATIN	360.34	50.8	0.41
DaH	MOL002259	Physciondiglucoside	608.6	41.65	0.63
DaH	MOL002268	Rhein	284.23	47.07	0.28
DaH	MOL002280	Torachrysone-8-O-beta-D-(6′-oxayl)-glucoside	480.46	43.02	0.74
DaH	MOL002281	Toralactone	272.27	46.46	0.24
DaH	MOL002297	Daucosterol_qt	386.73	35.89	0.7
DaH	MOL002251	Mutatochrome	552.96	48.64	0.61
DaH	MOL002260	Procyanidin B-5,3′-O-gallate	730.67	31.99	0.32
DaH	MOL002276	Sennoside E_qt	524.5	50.69	0.61
DaH	MOL002288	Emodin-1-O-beta-D-glucopyranoside	432.41	44.81	0.8
DaH	MOL002293	Sennoside D_qt	524.5	61.06	0.61
DaH	MOL002303	Palmidin A	510.52	32.45	0.65
DaH	MOL000472	Emodin^∗^	270.25	24.4	0.24
DaH	MOL001729	Crysophanol^∗^	254.25	18.64	0.21
DaH	MOL000476	Physcion^∗^	284.28	22.29	0.27
DiH	MOL002819	Catalpol^∗^	362.37	5.07	0.44
DiH	MOL003333	Acteoside^∗^	624.65	2.94	0.62
DiH	MOL000732	Stachyose^∗^	666.66	3.25	0.59
MDP	MOL000211	Mairin	456.78	55.38	0.78
MDP	MOL007374	5-[[5-(4-Methoxyphenyl)-2-furyl]`methylene]barbituric acid	312.3	43.44	0.3
MDP	MOL007369	4-O-methylpaeoniflorin_qt	332.38	67.24	0.43
MDP	MOL007382	Mudanpioside-h_qt 2	336.37	42.36	0.37
MDP	MOL007384	Paeonidanin_qt	330.41	65.31	0.35
SQ	MOL001494	Mandenol	308.56	42	0.19
SQ	MOL001792	DFV	256.27	32.76	0.18
SQ	MOL002879	Diop	390.62	43.59	0.39
SQ	MOL005344	Ginsenoside rh2	622.98	36.32	0.56
SQ	MOL007475	Ginsenoside f2	785.14	36.43	0.25
SQ	MOL007476	Ginsenoside rb1^∗^	1,109.46	6.29	0.04
SQ	MOL005338	Ginsenoside Re^∗^	947.3	4.27	0.12
SQ	MOL005341	Sanchinoside C1^∗^	801.14	10.04	0.28
SQ	MOL007487	Notoginsenoside r1^∗^	933.27	5.43	0.13
SCP	MOL001944	Marmesin	246.28	50.28	0.18
SCP	MOL003542	8-Isopentenyl-kaempferol	354.38	38.04	0.39
SCP	MOL003576	(1R,3aS,4R,6aS)-1,4-bis(3,4-dimethoxyphenyl)-1,3,3a,4,6,6a-hexahydrofuro[4,3-c]furan	386.48	52.35	0.62
SCP	MOL003578	Cycloartenol	426.8	38.69	0.78
MDP/SQ	MOL000098	Quercetin	302.25	46.43	0.28
CS/DaH/SQ	MOL000358	Beta-sitosterol	414.79	36.91	0.75
CS/DiH/MDP	MOL000359	Sitosterol	414.79	36.91	0.75
MDP/SCP	MOL000422	Kaempferol	286.25	41.88	0.24
CS/DiH/SQ	MOL000449	Stigmasterol	412.77	43.83	0.76
CS/MDP	MOL000492	(+)-Catechin	290.29	54.83	0.24
CS/MDP	MOL000874	Paeonol^∗^	166.19	28.79	0.04
CS/MDP	MOL001925	Paeoniflorin_qt	318.35	68.18	0.4
CS/MDP	MOL007003	Benzoyl paeoniflorin	584.62	31.14	0.54
DL	——	Xanthine	——	——	——
DL	——	Guanosine	——	——	——
DL	——	Xanthinin	——	——	——
DL	——	Hyrcanoside	——	——	——
SNJ	——	Arginine	——	——	——
SNJ	——	Aspartic acid	——	——	——
SNJ	——	Alanine	——	——	——
DL/SNJ	——	4-Guanidino-1-butanol	——	——	——
DL/SNJ	——	Cholesterol	——	——	——
DL/SNJ	——	Guanidine	——	——	——

“^∗^” means the active ingredients retrieved by literature mining that cannot fit the earlier screening criteria but with important pharmacological effects. “——” means the active ingredients collected from the BAT-MAN database with no ADME parameters. SNJ: *Cornu Bubali*; DaH: *Rheum palmatum* L.; DiH: *Rehmannia glutinosa* Libosch.; SQ: *Panax notoginseng* (Burk.) F.H.Chen; CS: *Radix paeoniae rubra*; MDP: Moutan Cortex; DL: *Pheretima aspergillum*; SCP: *Acori Tatarinowii Rhizoma*.

**Table 2:** Key nodes of component-target network.

Herb	Node	Degree	Betweenness centrality
MDP/SQ	Quercetin	112	0.36958975
SNJ	Alanine	104	0.41797809
MDP/SCP	Kaempferol	39	0.06875802
DaH	Emodin	29	0.04544646
DL/SNJ	Guanidine	27	0.08907938
DL	Xanthinin	25	0.08396791
DL	Xanthine	24	0.08789741
CS	Baicalein	23	0.02318204
CS/MDP	Paeonol	20	0.02429964
CS/DaH/SQ	Beta-sitosterol	20	0.02791937
DL	Guanosine	19	0.06005265
SCP	8-Isopentenyl-kaempferol	18	0.02608771
DaH	Aloe-emodin	18	0.01960079
CS/DaH/SQ	Stigmasterol	18	0.03894191
DL/SNJ	Cholesterol	16	0.05001097
CS	Ellagicacid	15	0.01790131
SNJ	Aspartic acid	13	0.03120851
SCP	Marmesin	13	0.0165369
DL/SNJ	4-Guanidino-1-butanol	12	0.03705792
DaH	EUPATIN	12	0.01168363
CS/MDP	Benzoylpaeoniflorin	12	0.01694236
CS	Albiflorin_qt	12	0.0149345
SQ	Ginsenoside rb1	11	0.01531869
SQ	Ginsenoside rh2	10	0.01552107
CS	1-O-beta-d-glucopyranosyl-8-o-benzoylpaeonisuffrone_qt	10	0.00756521
CS	Evofolin B	10	0.01443392
SQ	Notoginsenoside r1	9	0.00950678
SQ	Ginsenoside f2	9	0.0087669
DaH	Mutatochrome	9	0.0191967
DaH	Procyanidin B-5,3′-O-gallate	9	0.02300249
CS	Albiflorin	9	0.00983057
DiH	Stachyose	8	0.00668771
DaH	Palmidin A	6	0.010557
CS	8-Debenzoylpaeonidanin	4	1.33*E*-02
CS	Paeoniflorin	4	0.00892285
Target	PTGS2	24	0.03392333
Target	PTGS1	21	0.0243207
Target	ESR1	17	0.04743098
Target	MAPT	15	0.02405041
Target	PRKACA	15	0.0125587
Target	AR	14	0.0546651
Target	PTAFR	14	0.01000789
Target	PGR	13	0.04286906
Target	SLC6A2	13	0.01380599
Target	SLC6A3	12	0.00778408
Target	ADRB2	9	0.06270829
Target	BAX	8	0.01594706
Target	ESR2	8	0.01052083
Target	F10	8	0.0115522
Target	PRKCA	8	0.07827366
Target	RXRA	8	0.01249876
Target	TOP2A	8	0.01135885
Target	CDK2	7	0.00778363
Target	F7	7	0.00821191
Target	TNF	7	0.01279629
Target	F2	6	0.00701019
Target	IL1B	6	0.06205608
Target	NOS2	6	0.01729864
Target	PRKCD	6	0.00681975
Target	VEGFA	6	0.01754771
Target	AKR1B1	5	0.02396875
Target	JUN	5	0.02537208
Target	MMP9	5	0.00770693
Target	NOS3	5	0.02534139
Target	NR3C2	5	0.0069026
Target	STAT1	5	0.01728485
Target	ADORA1	4	0.04165433
Target	ADRA2A	4	0.01897542
Target	CYP1A1	4	0.01430838
Target	GSTP1	4	0.02559893
Target	HIF1A	4	0.02506214
Target	PPARD	4	0.02353186
Target	TGFB1	4	1.03*E*-02
Target	VDR	4	8.61*E*-03

**Table 3:** Number of genes expressed in each group of RNA-seq data.

Group	Total genes	Differently expressed genes	Upregulated genes	Downregulated genes
CGT vs. CG	29049	613	324	289
MG vs. CG	29049	766	314	452
MGT vs. MG	29049	583	308	275

CG: control group; CGT: the control group with LXTYF treatment; MG: ICH-model group; MGT: AICH model with LXTYF-treatment group.

**Table 4:** KOBAS pathway enrichment results of 583 differentially expressed mRNAs.

Pathway name	ID	Input number	Background number	*p* value	Corrected *p* value
Metabolic pathways	hsa01100	37	1243	4.87*E*-06	0.000776
MAPK signaling pathway	hsa04010	11	255	0.000719	0.022949
Calcium signaling pathway	hsa04020	11	180	4.07*E*-05	0.004326
Oxytocin signaling pathway	hsa04921	9	158	0.00033	0.014855
Regulation of actin cytoskeleton	hsa04810	9	215	0.002595	0.063668
Endocytosis	hsa04144	9	260	0.008413	0.107348
Apoptosis	hsa04210	8	140	0.000686	0.022949
Focal adhesion	hsa04510	8	203	0.006202	0.104136
Neuroactive ligand-receptor interaction	hsa04080	8	278	0.032413	0.198843
Circadian entrainment	hsa04713	7	95	0.000356	0.014855
TNF signaling pathway	hsa04668	7	110	0.000812	0.02355
cGMP-PKG signaling pathway	hsa04022	7	167	0.007476	0.106674
cAMP signaling pathway	hsa04024	7	199	0.017613	0.156542
Tight junction	hsa04530	6	139	0.011322	0.124199
RNA degradation	hsa03018	5	77	0.004147	0.094488
Phosphatidylinositol signaling system	hsa04070	5	98	0.010704	0.121948
Leukocyte transendothelial migration	hsa04670	5	118	0.02151	0.171545
AMPK signaling pathway	hsa04152	5	125	0.026528	0.188053
Dopaminergic synapse	hsa04728	5	130	0.030533	0.1948
Cell adhesion molecules (CAMs)	hsa04514	5	146	0.045802	0.231919
Arginine and proline metabolism	hsa00330	4	50	0.005113	0.10411
Lysine degradation	hsa00310	4	52	0.005821	0.10411
Inositol phosphate metabolism	hsa00562	4	71	0.015938	0.154071
mRNA surveillance pathway	hsa03015	4	92	0.035408	0.200721
Fc gamma R-mediated phagocytosis	hsa04666	4	93	0.036571	0.201138
HIF-1 signaling pathway	hsa04066	4	103	0.049393	0.242404
Pyruvate metabolism	hsa00620	3	40	0.017749	0.156542
Cysteine and methionine metabolism	hsa00270	3	45	0.023739	0.180305
Nucleotide excision repair	hsa03420	3	47	0.026404	0.188053
Notch signaling pathway	hsa04330	3	48	0.027794	0.18808
Fanconi anemia pathway	hsa03460	3	55	0.038589	0.206572
Arginine biosynthesis	hsa00220	2	21	0.034827	0.200721
Glycosylphosphatidylinositol(GPI)-anchor biosynthesis	hsa00563	2	25	0.046729	0.232917

**Table 5:** Details of connections of important nodes in the H-C-T-P network.

Connections	Node 1	Node 2
Herb-Cpmpound	CS	(+)-Catechin, (2R,3R)-4-methoxyl-distylin, 1-o-beta-d-glucopyranosyl-8-o-benzoylpaeonisuffrone_qt, 1-o-beta-d-glucopyranosylpaeonisuffrone_qt, baicalein, benzoylpaeoniflorin, beta-sitosterol, evofolin B, isobenzoylpaeoniflorin, lactiflorin, paeoniflorigenone, paeoniflorin_qt, paeonol, 4-ethyl-paeoniflorin_qt, 4-o-methyl-paeoniflorin_qt, 8-debenzoylpaeonidanin, 9-ethyl-neo-paeoniaflorinA_qt, albiflorin, albiflorin_qt, stigmasterol
Herb-Cpmpound	DaH	(-)-Catechin, aloe-emodin, beta-sitosterol, crysophanol, emodin, EUPATIN, mutatochrome, physcion, rhein, toralactone
Compound-target	1-O-beta-d-glucopyranosyl-8-o-benzoylpaeonisuffrone_qt, 1-o-beta-d-glucopyranosylpaeonisuffrone_qt, 4-ethyl-paeoniflorin_qt, 4-O-methylpaeoniflorin_qt, 4-o-methyl-paeoniflorin_qt, 9-ethyl-neo-paeoniaflorinA_qt, albiflorin, albiflorin_qt, benzoylpaeoniflorin, ginsenosidef2, isobenzoylpaeoniflorin, lactiflorin, mutatochrome, paeoniflorigenone	PTAFR
Compound-target	Aloe-emodin, baicalein, beta-sitosterol, catalpol, emodin, ginsenosiderh2, kaempferol, quercetin	CASP3
Target-pathway	CACNA1H, CASP3, DUSP1, FGF14, FOS, GADD45G, MEF2C, NLK, NR4A1, SRF, TAOK2	MAPK signaling pathway
Target-pathway	CACNA1H, PTAFR, DRD1, NOS1, NOS2, PHKA2, RYR2, RYR3, SLC8A2, VDAC1, VDAC3	Calcium signaling pathway

## Data Availability

The chemical ingredients of six botanical medicinal materials in LXTYF were obtained from Traditional Chinese Medicine Systems Pharmacology Database and Analysis Platform (TCMSP) (http://lsp.nwu.edu.cn/tcmsp.php) The chemical ingredients of the other two animal medicines in LXTYF were derived from BATMAN-TCM (http://bionet.ncpsb.org/batman-tcm). Different genes linked to ICH were collected from three existing resources with Medical Subject Heading (MeSH) “Cerebral Hemorrhage”: (1) GAD (https://geneticassociationdb.nih.gov/), (2) Genecards (https://www.genecards.org/), and (3) DisGeNET (http://www.disgenet.org/web/DisGeNET).

## Supplementary Materials

Supplementary Materials S1 Table. 517 compounds of 8 herbs in LXTYF collected from TCMSP and BATMAN-TCM database. S2 Table. 874 targets of the active ingredients obtained from the TCMSP. S3 Table. 149 targets of active components predicted from the SwissTargetPrediction database. S4 Table. 2315 targets relating to ICH collected from GAD, Genecards, and DisGeNET database. S5 Table. Topological parameters of component-target network. S6 Table. Proteins-proteins interaction information of LXTYF targets for AICH in STRING 11.0. S7 Table. Topological parameters of the PPI network. S8 Table. Results of putative targets for KEGG pathway enrichment analysis. S9 Table. Detailed information of 583 differentially expressed mRNAs identified between MGT and MG. S10 Table. Details of the Herb-Compound-Target-Pathway(H-C-T-P) network connection verified in RNA-seq.
